# Supplementary material for: Dissociable memory modulation mechanisms facilitate fear amnesia at different timescales
Source: eLife. 2025 Dec 15;13:RP98652. doi: 10.7554/eLife.98652 (PMC12705067; doi:10.7554/eLife.98652)
Supplement: Supplementary file 1. [file elife-98652-supp1.docx]

**Supplementary File 1**. Participant exclusion criteria.

|  | **Total subject #** | **Non-responder** | **Non-learner** in **Acquisition** | **Non-learner** in **Extinction** | **Final subject #** |
| --- | --- | --- | --- | --- | --- |
| Exclusion criterion |  | Mean CS SCR < 0.02 u*S* | 1. Mean CS+ SCR < CS- SCR in the latter half trials AND 2. diff SCR (CS+, CS-) in the 2^nd^ half trials **<** diff SCR (CS+, CS-) the 1^st^ half trials | 1. Mean CS+ SCR > CS- SCR in the latter half and last trials) AND 2. diff SCR (CS+, CS-) 2^nd^ half trials **>** diff SCR (CS+, CS-) the 1^st^ half trials |  |
| Study 1 (reminder group) | 36 | 0 | 3 | 3 | 30 |
| Study 1 (no-reminder group) | 39 | 8 | 1 | 3 | 27 |
| Study 2 (30min group) | 48 | 19 | 1 | 1 | 27 |
| Study 2 (6hr group) | 31 | 5 | 0 | 0 | 26 |
| Study 2 (24hr group) | 40 | 13 | 1 | 0 | 26 |
| Study 3(R-PFC) | 22 | 3 | 0 | 0 | 19 |
| Study 3 (R-VER) | 22 | 4 | 0 | 0 | 18 |
| Study 3 (NR-PFC) | 22 | 4 | 0 | 0 | 18 |
| Study 3(NR-VER) | 24 | 4 | 0 | 0 | 20 |
